# The Clinicopathologic and Prognostic Value of Altered Chromosome 17 Centromere Copy Number in HER2 Fish Equivocal Breast Carcinomas

**DOI:** 10.1371/journal.pone.0132824

**Published:** 2015-07-10

**Authors:** Hongfei Ji, Qijia Xuan, Abiyasi Nanding, Haiyu Zhang, Qingyuan Zhang

**Affiliations:** 1 Department of Cancer Molecular and Biology, Cancer Institute of Harbin Medical University, Harbin, China; 2 Department of Medical Oncology, Tumor Hospital of Harbin Medical University, Harbin, China; 3 Department of Pathology, Tumor Hospital of Harbin Medical University, Harbin, China; 4 Department of Biostatistics, Harbin Medical University, Harbin, China; University Medical Center Hamburg-Eppendorf, GERMANY

## Abstract

Chromosome 17 centromere (CEP17) gain is frequently observed in breast cancer by fluorescence in situ hybridization (FISH). To address the biologic characteristics and clinical significance of CEP17 gain in a large population of breast cancer patients, we performed FISH on a series of 770 breast cancer tissues to evaluate the status of human epidermal growth factor receptor 2 (HER2) gene and CEP17 by immunohistochemistry (IHC) and FISH. Among the 770 specimens, 184 cases showed CEP17 gain (23.9%). Histological grade, nodal status, HER2 by IHC, Ki 67 index, and p53 expression were significantly different between CEP17 gain tumors and HER2-positive tumors. In contrast with HER2-negative tumors, CEP17 gain tumors showed higher histological grade, higher HER2 score by IHC, and higher Ki 67 index. The patients with CEP17 gain tumors had an intermediate survival between HER2-negative and HER2-positive patients. By comparison to HER2-negative and HER2-positive patients, survival in luminal B patients with CEP17 gain tumors also fell in between. In conclusion, CEP17 gain tumors show specific differences compared with HER2-negative and HER2-positive tumors in clinical parameters and prognosis.

## Introduction

Breast cancer is one of the most common cancers in women and one of the leading causes of death among women [1–3]. Human epidermal growth factor 2 (HER2), which is located on the long arm of chromosome 17 (17q12-21-21.32), is an important oncogene in breast cancer [4–5]. Many studies have demonstrated overexpression and amplification of the HER2 transmembrane tyrosine kinase receptor in approximately 20–30% of breast cancer cases, and this is associated with not only poor clinical outcome, but also response to clinical therapies [6–8]. Therefore, it is crucial to accurately determine HER2 status in breast cancer patients for selecting the appropriate therapeutic regimen.

Fluorescence in situ hybridization (FISH) and immunohistochemistry (IHC) are the most common testing methods and commonly used for the assessment of HER2 status. Although sufficient evidence indicates that FISH and IHC can accurately and efficiently define HER2 positivity in breast cancer, some specimens show ambiguous HER2 results, which are mostly due to CEP17 gain, a common genotypic abnormality [9–12]. Few studies has specifically examined CEP17 status and compared differences of potential effects, clinical parameters, and prognosis with HER2-negative status and-positive status. The aim of the present study was to analyze the biologic characteristics and clinical significance of CEP17 gain and explore the impact on luminal B molecular subclassification combined with estrogen receptor (ER) and progesterone receptor (PR) expression in a large population of breast cancer patients.

## Materials and Methods

### Ethics Statement

This study, including the procedures for patient enrollment and recruitment, was approved by the Institutional Review Board of the Affiliated Tumor Hospital of Harbin Medical University, and all patients who participated in the study provided written informed consent.

### Patient samples

We evaluated the tissues of patients with invasive breast cancer that showed equivocal or positive HER2 status on IHC. Patient samples were analyzed by FISH from 1/1/2007 to 5/30/2009 in the Tumor Hospital of Harbin Medical University. We excluded the patients who were with metastatic lesions and receiving non-standard treatment. The remaining 670 patients and the 100 patients with negative HER2 status on IHC were enrolled in the study. The date of surgery was used to represent the beginning of the follow-up period and follow-ups were terminated in May 2014. Patients who developed recurrence or metastasis were identified during the follow-ups by tumor marker measurements and adequate diagnostic imaging modalities.

### Methods

#### IHC

**Fig 1:**
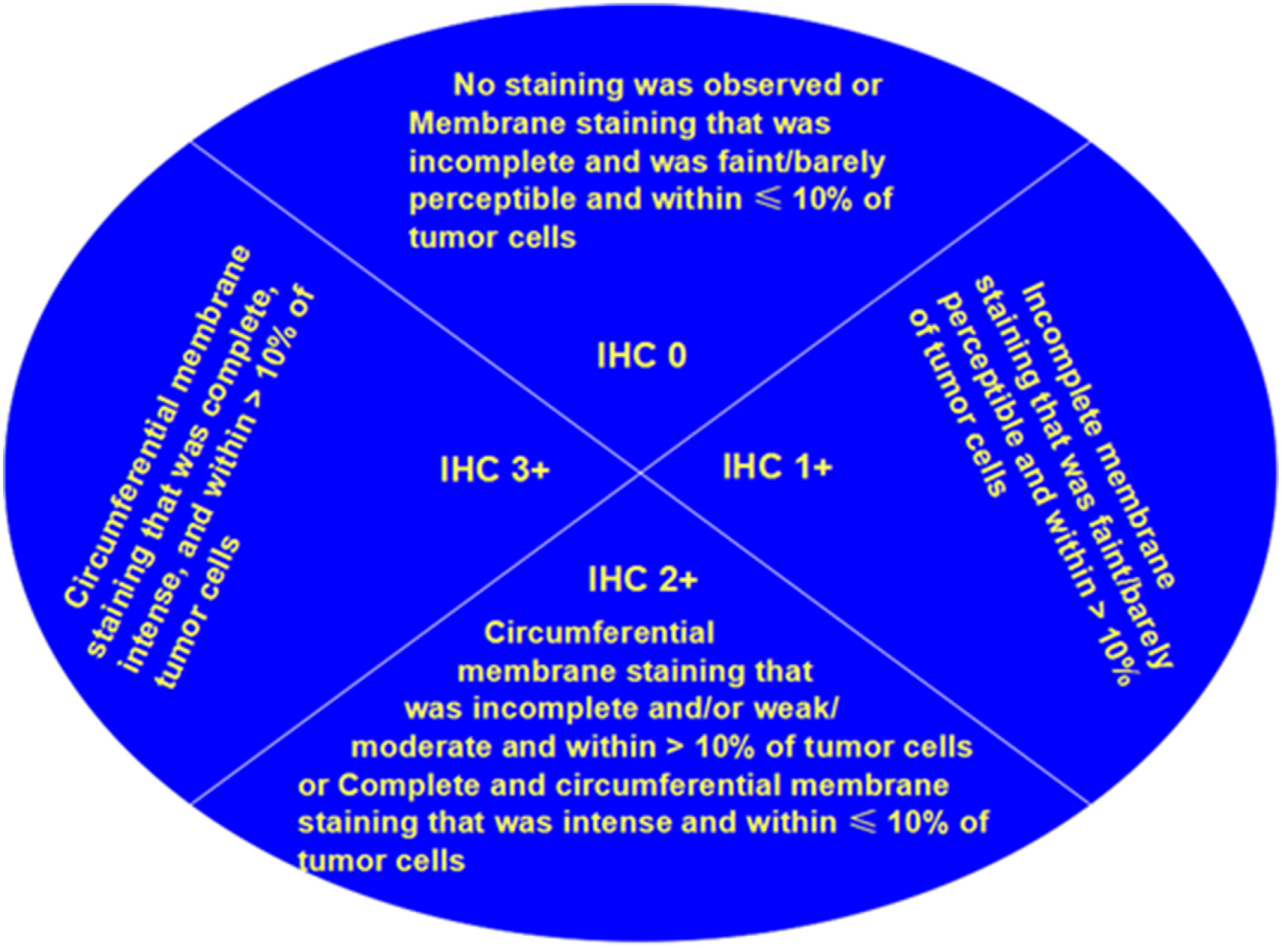
Classification of evaluation of HER2 protein expression by IHC of the invasive component of a breast cancer specimen.

All breast cancer tissues were formalin-fixed and paraffin-embedded. For each specimen, a 4-micrometers-thick tissue section was provided on a slide. The immunohistochemical analysis was executed as depicted previously [13]. In brief, slides were incubated with HER2 polyclonal antibody (Dako, California, USA), P53 (1:500, DO-7; Dako), Ki-67 (1:300, MIB-1; Dako). The stained tissues were evaluated according to the densities of staining and the number of stained cells. Tissues were considered positive for Ki-67 with more than 20% stained of the cells examined. Any staining of cells was deemed as P53 positive. The staining of cell membrane was considered HER2 protein expression. The slides have been reevaluated by two pathologists to assess HER2 status following the 2013 ASCO/CAP recommendations (Fig 1) [10]. The positive controls were the sections with strong membrane staining. Each section was scanned at ×100 and×400 magnification by microscope (Olympus BX51).

#### FISH

FISH analysis was performed on paraffinized 5-micrometers-thick sections using the Vysis LSI HER2 SpectrumOrange and CEP17 SpectrumGreen Dual Color DNA probe kit (VysisPath Vysion, Abbot Laboratories, Illinois, USA) in line with the manufacturer’s instructions. In short, pretreated procedure comprised the following steps: sections baking, deparaffin, dehydration, deproteinization and refixation. Then, according to the protocol, the sections were denatured and hybridized hybridization oven. Finally the sections were washed, counterstained with 4',6-Diamidino-2-Phenylindole (DAPI, invitrogen, NY, USA) for signal calculation. Scoring of HER2 and CEP17 probe signals were achieved by a fluorescence microscope (Olympus BX51). The slides have been reevaluated by two independent and certified pathologists and with identified the invasive area. The pathologists assessed HER2 status following the 2013 ASCO/CAP recommendations [10]. FISH results were interpreted depending on two different scoring methods: (1) based on absolute HER2 gene copy number or (2) based on the ratio of HER2 gene/CEP17 copy number (HER2/CEP17 ratio). As recommended by the ASCO/CAP guidelines, a HER2/CEP17 ratio of less than 2.0 with an average HER2 copy number less than 4.0 signals/cell was considered HER2 negative, and a HER2/CEP17 ratio of higher than or equal to 2.0 or an HER2/CEP17 ratio of less than 2.0 with an average HER2 copy number higher than 6.0 signals/cell was considered HER2 positive. CEP17 gain was defined as an average CEP17 copy number between 3.76 and 6.

### Statistical methods

The Chi-square test were used for comparing clinicopathologicfeatures between tumor groups. The time of disease-free survival was defined as the time between diagnosis and local recurrence, distant metastasis, or death from breast cancer. The survival curves were analyzed by the Kaplan–Meier method and the comparison between curves was evaluated by the log-rank test. All the statistical analyses were accomplished by the IBM SPSS statistics version 20.0 (SPSS Inc. Illinois, USA). Statistical significance was determined as p-values <0.05.

## Results

### Clinicopathologic characteristics of CEP17 gain tumors

**Fig 2:**
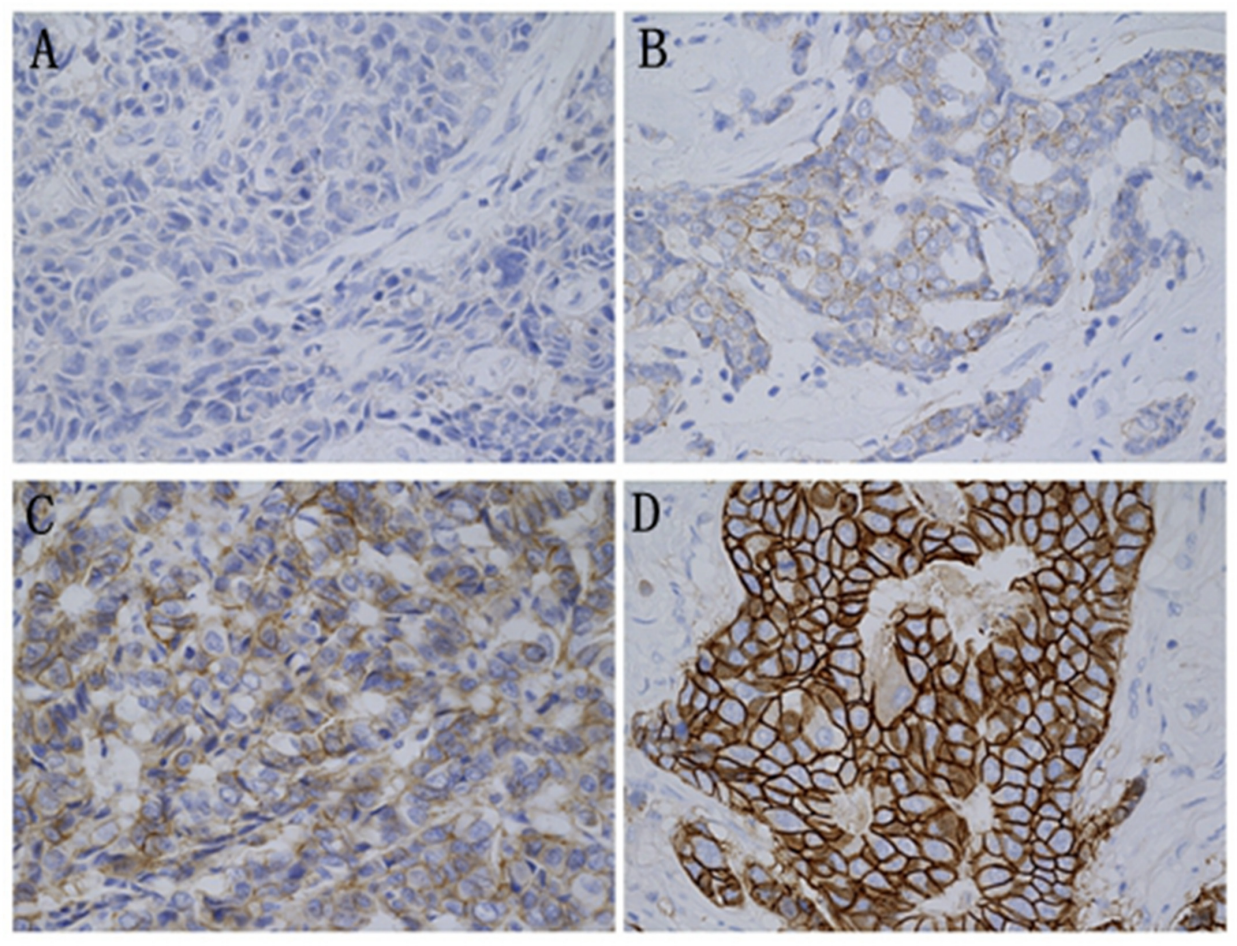
HER2 protein expression detected by IHC. (×400) A: Negative (score 0). B: Negative (score 1+). C: Equivocal (score 2+). D: Positive (score 3+).

**Fig 3:**
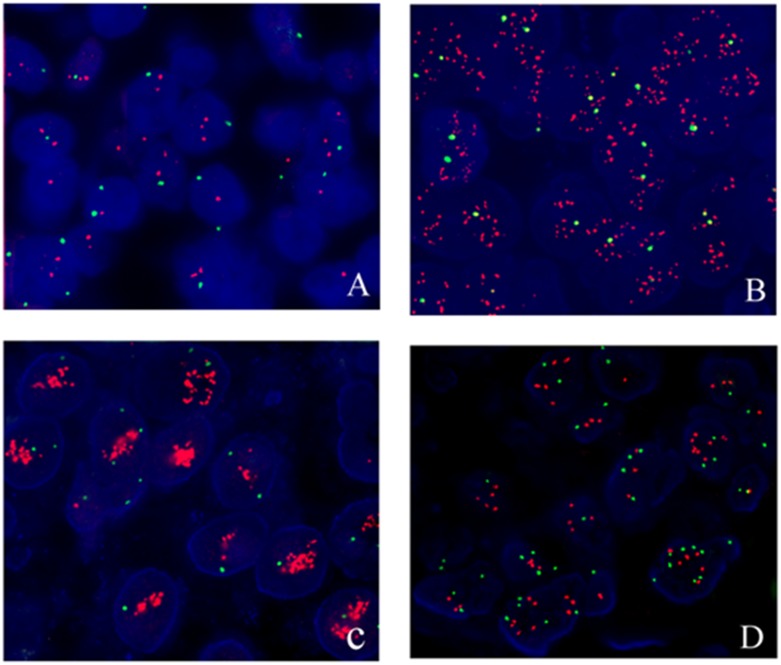
HER2 gene status identified by FISH. Red signals represent HER2 gene, green signals represent CEP17 (×600). A: HER2 gene negative case; B: HER2 gene positive case, HER2/CEP17 ratio of higher than 2.0; C: HER2 gene positive case, HER2 signals are clustered; D: HER2 gene with CEP17 gain, average CEP17 copy number ≥3.76 to 6.

Representative images of evaluations of HER2 expression by IHC and FISH, respectively, are presented in Figs 2 and 3. Among the 770 specimens studied by IHC and FISH, 184 cases showed CEP17 gain (23.9%). The patients were divided into 3 groups by HER2 results on FISH (HER2-negative, HER2-positive and CEP17 gain). Table 1 shows the clinicopathologic parameters of the patients in the 3 groups. In contrast with HER2-negative tumors, CEP17 gain tumors presented higher histological grade (*P* = 0.042), higher HER2 score by IHC (*P*<0.001), and higher Ki67 index (*P* = 0.030). Histological grade (*P*<0.001), nodal status (*P*<0.001), HER2 score by IHC (*P*<0.001), Ki 67 index (*P*<0.001), and P53 expression (*P* = 0.003) were strikingly different between HER2-positive tumors and CEP17 gain tumors. By comparison with HER2-negative tumors, the HER2-positive tumors expressed higher histological grade (*P* = 0.026), more lymph mode metastasis (*P*<0.001), higher HER2 score by IHC (*P*<0.001), higher Ki 67 status (*P*<0.001) and higher frequency of P53 positive status (*P*<0.001). Taken together, these data indicate that CEP17 gain tumors are significantly different from HER2-negative and HER2-positive tumors.

**Table 1:** Distribution of clinicopathologic features in CEP17 gain tumors compared with HER2–negative and HER2–positive tumors defined by FISH.

Characteristic	HER2-Negative			CEP17Gain			HER2-Positive		
	(n = 250)			(n = 184)			(n = 336)		
	No	%	P^1^	No	%	P^2^	No	%	P^3^
**Age (years)**			0.238			0.164			1.000
≤ 50 years	151	60.4		100	54.3		204	60.7	
> 50 years	99	39.6		84	45.7		132	39.3	
**Tumor size(cm)**			0.331			0.197			**0.008**
≤ 2 cm	136	54.4		91	49.5		145	43.2	
> 2 cm	114	45.6		93	50.5		191	56.8	
**Histological-grade**			**0.042**			**0.000**			**0.026**
I	13	5.2		18	9.8		6	1.8	
II	217	86.8		143	77.7		290	86.3	
III	20	8		23	12.5		40	11.9	
**Nodal status**			0.175			**0.000**			**0.000**
N0	135	54		87	47.3		85	25.3	
N+	115	46		97	52.7		251	74.7	
**Menopause status**			0.135			0.780			0.198
Premenopause	162	64.8		106	57.6		199	59.2	
Postmenopause	88	35.2		78	42.4		137	40.8	
**BMI**			0.790			0.071			0.100
< 28	212	84.8		154	83.7		301	89.6	
≥28	38	15.2		30	16.3		35	10.4	
**ER status**			0.308			0.397			0.831
ER+	202	80.8		156	84.8		274	81.5	
ER-	48	19.2		28	15.2		62	18.5	
**PR status**			0.406			0.612			0.103
PR+	165	66		129	70.1		243	72.3	
PR-	85	34		55	29.9		93	27.7	
**HER2 by IHC**			**0.000**			**0.000**			**0.000**
0/1+	100	40		20	10.9		14	4.2	
2+	143	57.2		148	80.4		158	47	
3+	7	2.8		16	8.7		164	48.8	
**Ki 67status**			**0.030**			**0.000**			**0.000**
<20%	158	63.2		97	52.7		115	34.2	
≥20%	92	36.8		87	47.3		221	65.8	
**P53 status**			0.742			**0.003**			**0.000**
Positive	65	26		51	27.7		138	41.1	
Negative	185	74		133	72.3		198	58.9	

^1^ HER2-negative versus CEP17 gain.

^2^ CEP17 gain versus HER2- positive.

^3^ HER2-positive versus HER2-negative.

### Survival curves characteristics of the CEP17 gain patients

**Fig 4:**
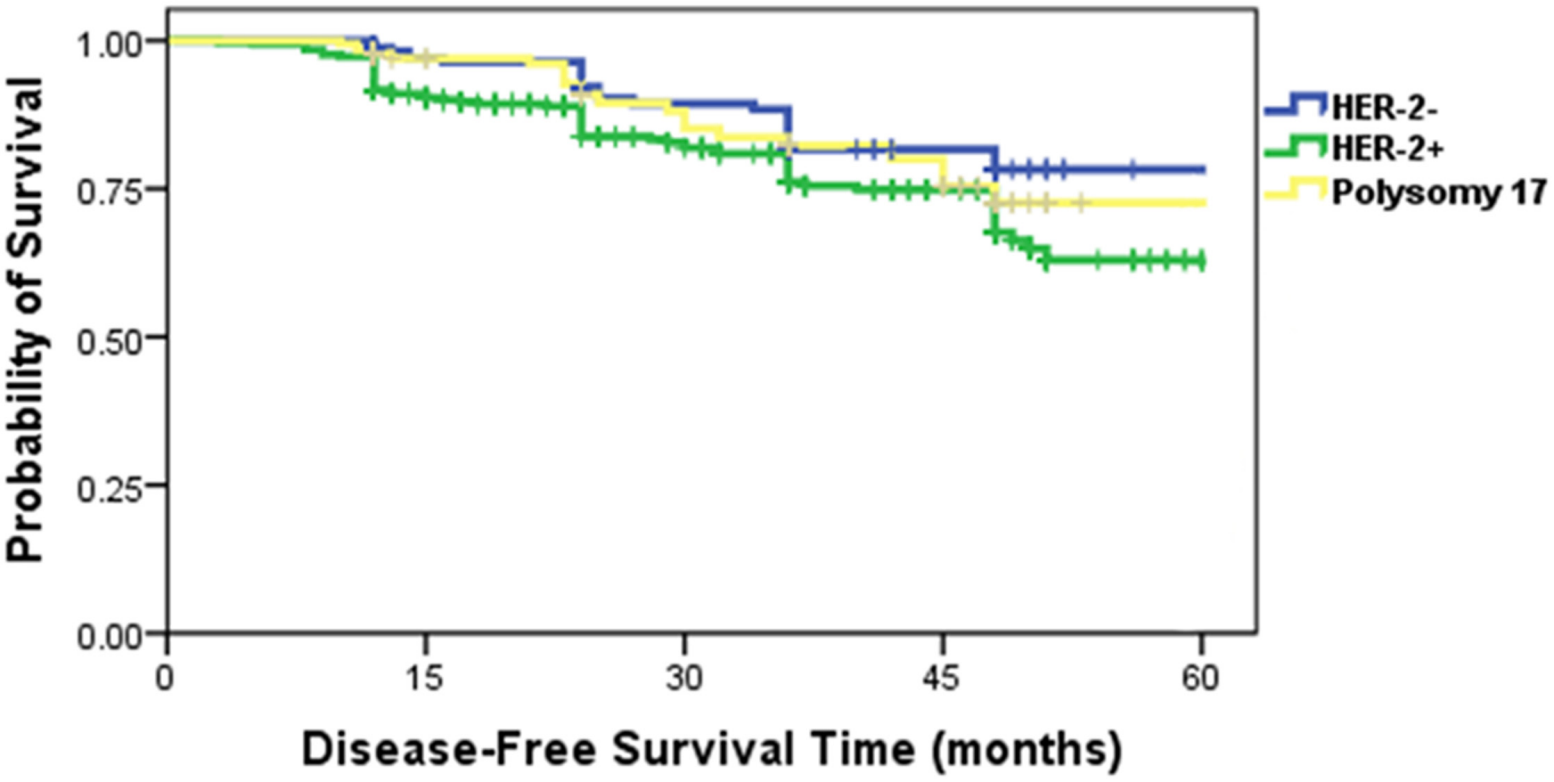
Kaplan–Meier analysis of disease-free survival time in the enrolled patients.

We preformed Kaplan–Meier survival analysis in the three groups (CEP17 gain, HER2- negative and HER2-positive patients). And the result demonstrated that disease-free survival in patients with CEP17 gain tumors is shorter than HER2-negative patients, and longer compared with HER2-positive patients. The patients with CEP17 gain tumors had an intermediate survival (*P* = 0.014) (Fig 4).

### Survival curves characteristics of CEP17 gain in the luminal B patients

**Fig 5:**
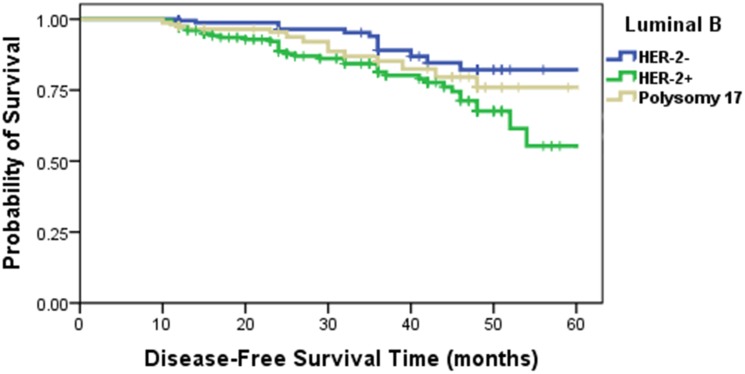
Kaplan–Meier analysis of disease-free survival time in the luminal B patients.

The characteristics of luminal B tumors present ER/PR positive, HER2 negative and Ki67 index ≥14% or ER/PR positive and HER2 positive on IHC. Despite expressing ER, the luminal B subtype confers increased risk of early relapse with endocrine therapy compared with the luminal A subtype [14–16]. We divided the luminal B patients into three subgroups by HER-2 signal number. HER2-positive, HER2-negative and patients with CEP17 gain tumors. Among the luminal B patients, Kaplan–Meier survival curves (Fig 5) illustrated different disease-free survival in three subgroups patients., the survival time in patients with CEP17 gain tumors was between (*P* = 0.025) HER2-negative and HER2-positive patients.

## Discussion

Breast cancer, one of heterogeneous diseases, is categorized into four major subtypes based on the breast cancer-related receptors ER, PR, and HER2: luminal A, luminal B, HER2+/ER−, and basal-like [15–16]. These subtypes exhibit distinct differences in a variety of aspects, including genetic alterations, clinical features, responses to specific chemotherapy, and prognosis [16–18]. Therefore, accurate assessment of ER, PR, and HER2 status is a key point for the classification of breast cancer. Several limitations and disadvantages in the identification of HER2 status compared with ER and PR still exist. As a consequence, a portion of breast cancer patients have been determined as equivocal status instead of specifically positive or negative status.

A growing body of recent studies suggested that CEP17 gain is the major mechanism underlying HER2 equivocal status [18–24]. The CEP17 probe was used to measure the copy number of CEP17. Several different thresholds (between two and four copies per cell) have been used regarding CEP17 gain because of a lack of official guidelines [25–26]. Based on these varying thresholds, the frequency of CEP17 gain in breast cancer specimens varies from 13% to 46% [27–29]. In this study, the threshold for CEP17 gain was used 3.76 to 6 copies, and 23.9% specimens (184 in 770) were observed to harbor CEP17 gain.

There are several reasons for choosing the threshold of 3.76 to 6 copies per cell. Firstly, abnormality of CEP17 number is generally can be divided into, CEP17 low polysomy (2.26 to 3.75 signals per cell), and CEP17 high polysomy (3.76 to 6 signals per cell) [30–31]. According to published reports, specimens with less than 3.75 copies (hypodisomy and low polysomy) were close to disomy. And in the high polysomy and low polysomy patients, HER2 protein expression was significantly different [32–34]. Furthermore, this threshold will confer a precise definition CEP17 gain to the specimens identified as HER2 equivocal status [10]. Using the threshold, these equivocal specimens would be stratified into the CEP17 gain status and HER2-negative status. Most importantly, our results suggest that the CEP17 gain tumors are significantly different from HER2-negative and HER2-positive tumors in severity-related parameters. For example, histological grade, Ki67 index, nodal status, and p53 expression. In addition, survival analysis indicated that the survival rates in CEP17 gain and HER2-negative and HER2-positive subgroups were significantly different. When the luminal B patients were stratified into three subgroups by FISH results, the significance differences were also observed between the different subgroups. Previous studies have shown controversial results regarding the association between CEP17 gain and clinical parameters, and only a few studies could provide evidence about clinicopathologic value of chromosome gain 17 polysomy, specifically about prognosis [35–39]. We speculate that the lower threshold, along with genetic heterogeneity, may be responsible for such equivocal results.

In summary, we have investigated the frequency and clinical significance of CEP17 gain in 770 breast cancer specimens. Based on the threshold, CEP17 gain tumors are significantly different from HER2-negative and HER2-positive tumors. This study provides applicable implications for the definition of CEP17 gain that argue against the previous classification. Patients with CEP17 gain should be subtyped and can be distinguished from patients with HER2 gene amplification or no amplification. This study will help to determine the best therapeutic response to HER2-targeted therapy in breast cancer patients with CEP17 gain.
